# Technical support and delegation to practice staff – status quo and (possible) future perspectives for primary health care in Germany

**DOI:** 10.1186/1472-6947-12-81

**Published:** 2012-08-01

**Authors:** Elisabeth Urban, Dominik Ose, Stefanie Joos, Joachim Szecsenyi, Antje Miksch

**Affiliations:** 1 Department of General Practice and Health Services Research, University of Heidelberg Hospital, Heidelberg, Germany

**Keywords:** Primary care, Health information technology, Non-physician practice staff, Support, Delegation, Electronic prescribing, Electronic communication, Recall system, Reminder system

## Abstract

**Background:**

Primary health care in industrialized countries faces major challenges due to demographic changes, an increasing prevalence of chronic diseases and a shortage of primary care physicians. One approach to counteract these developments might be to reduce primary care physicians’ workload supported by the use of health information technology (HIT) and non-physician practice staff. In 2009, the U.S. Commonwealth Fund (CWF) conducted an international survey of primary care physicians which the present secondary descriptive analysis is based on. The aim of this analysis was twofold: First, to explore to what extend German primary care physicians already get support by HIT and non-physician practice staff, and second, to show possible future perspectives.

**Methods:**

The CWF questionnaire was sent to a representative random sample of 1,500 primary care physicians all over Germany. The data was descriptively analyzed. Group comparisons regarding differences in gender and age groups were made by means of Chi Square Tests for categorical variables. An alpha-level of p < 0.05 was used for statistical significance.

**Results:**

Altogether 715 primary care physicians answered the questionnaire (response rate 49%). Seventy percent of the physicians use electronic medical records. Technical features such as electronic ordering and access to laboratory parameters are mainly used. However, the majority does not routinely use technical functions for drug prescribing, reminder-systems for guideline-based interventions or recall of patients. Six percent of surveyed physicians are able to transfer prescriptions electronically to a pharmacy, 1% use email communication with patients regularly. Seventy-two percent of primary care physicians get support by non-physician practice staff in patient care, mostly in administrative tasks or routine preventive services. One fourth of physicians is supported in telephone calls to the patient or in patient education and counseling.

**Conclusion:**

Within this sample the majority of primary care physicians get support by HIT and non-physician practice staff in their daily work. However, the potential has not yet been fully used. Supportive technical functions like electronic alarm functions for medication or electronic prescribing should be improved technically and more adapted to physicians’ needs. To warrant pro-active health care, recall and reminder systems should get refined to encourage their use. Adequately qualified non-physician practice staff could play a more active role in patient care. Reimbursement should not only be linked to doctors’, but also to non-physician practice staff services.

## Background

Strengthening the primary care setting is an important goal within the German health care system. Primary care contributes to a more equal distribution of health care services within populations and can result in lower health care costs [1]. Internationally, there are great efforts to strengthen primary care and adapt it to recent health care requirements [2]. As in other countries, primary health care in Germany faces major challenges: The demographic development with a growing percentage of older people as well as the increasing prevalence of chronic diseases and multimorbidity contributes to an increasing demand for health care services. Nearly ninety percent of German primary care physicians see patients with multiple chronic diseases frequently [3]. On the other side, Germany like other western countries is confronted with a shortage in primary care physicians, especially in rural areas [4].

In 2009, the U.S. Commonwealth Fund (CWF) conducted a survey of primary care physicians in 11 countries. The survey revealed considerable discrepancies of the primary care physicians’ perspective in the different health care systems. Results of the survey in international comparison have already been published: In Germany, the proportion of doctors who are dissatisfied with their job and health care system is significantly higher than in other countries. Most of the respondents in Germany express the need for fundamental changes or even complete reforms of the health care system. For the majority of primary care physicians the time required for accounting, documentation of clinical information or for legal requirements or coordination of patient care is very problematic [5,6]. These findings confirm the results from previously conducted CWF-surveys in Germany [3].

In the context of growing health care requirements and a shortage of physicians in primary care, these findings paint a rather gloomy picture of the future of German primary health care. To counteract this development, concepts must be evolved to secure nationwide primary health care in the long term.

Health information technology (HIT) and electronic medical records (EMR) have the potential to support physicians by reducing administrative and organizational tasks. Furthermore, EMR offer the possibility to integrate electronic reminders or alerts. HIT might even have an effect on patient safety [7]. The implementation of an EMR in primary care can lead to reduced costs for example in drug expenditure [8]. Routine electronic communication with patients via email or telephone represents a new approach in patient care.

Additionally, primary care physicians can be supported by non-physician practice staff in medical tasks. In other countries, delegation of medical tasks or even substitution of doctors by non-physician practice staff is already more established than in Germany [9]. The shift from acute towards chronic diseases demands a change in health care management. Concepts like the chronic care model emphasize the provision of pro-active care by practice teams composed of doctors and non-physician practice staff [10-12].

The aim of the present secondary analysis of the CWF-data is twofold. The first aim is to explore to what extent German primary care physicians get support in daily routine by HIT or non-physician practice staff. The second aim is to identify possible actions for improvement and future perspectives to reduce the physicians’ workload.

## Methods

The Commonwealth Fund (CWF) regularly conducts international surveys of patients’ and physicians’ care experiences and ratings on dimensions of care. In 2009, 11 countries took part in a survey of primary care physicians: Australia, Canada, France, Germany, Italy, the Netherlands, New Zealand, Norway, Sweden, the United Kingdom, and the United States of America. The survey was carried out from February to July and was coordinated by Harris Interactive Inc. In all countries, either structured interviews by telephone were conducted or questionnaires were provided by mail or online. The original questionnaire was translated into the language of each country. Altogether, 10,320 interviews were conducted among primary care physicians.

In Germany, the survey was financed by the Institute for Quality and Efficiency in Health Care (Institut für Qualität und Wirtschaftlichkeit im Gesundheitswesen, IQWiG) and conduced and coordinated by the Department of General Practice and Health Services Research at the University Hospital Heidelberg. For this survey a representative sample of 1,500 primary care physicians in Germany was randomly chosen. The distribution of physicians within all Federal States of Germany was adapted from data of the National Association of Statutory Health Insurance Physicians (Kassenärztliche Bundesvereinigung, KBV). The participants of the study in Germany were general practitioners as well as specialists for internal medicine and for pediatrics working in primary care. As an incentive, all participating physicians received 20 Euros which could optionally be donated to the organization Médecins Sans Frontières. To increase the response rate, two reminders were sent out during the following six weeks after the first contact. Non-responders were asked to return at least a postcard with sociodemographic data and a general judgment about the German health care system. Details of the data acquisition have already been published [5].

All data in this study were categorical variables and were therefore analyzed as frequencies and percentages. Results were displayed descriptively. All statistical analyses were conducted with SPSS version 18.0 (SPSS Inc., Chicago IL, USA). Group comparisons regarding differences in gender and age groups (up to the age of 49 years and age of 50 years and over) were made by means of Chi Square Tests for categorical variables. An alpha-level of p < 0.05 was considered statistically significant. However, as this was an exploratory analysis, p-values can only be descriptive in nature and therefore should be interpreted carefully. Deriving causal links from these findings should be made with caution.

## Results

In Germany, out of a sample of 1,500 primary care physicians, 715 physicians answered the questionnaire (response rate 49%). This response rate was above average in comparison to all other countries [5]. Forty-nine letters were returned because of invalid post addresses. Moreover 123 physicians (9%) did not answer the questionnaire, but sent back the non-responder postcard.

### Respondents

Regarding to gender, federal state and medical specialization, the sample differed only minimally compared to data from the KBV, that means a deviation not exceeding 3% per category. A comparison of the sample with non-responders revealed no significant differences regarding age, gender, location of practice, working experience of physicians, working in a multidisciplinary team, or overall satisfaction with the health care system [6].

Tables 1 and 2 show the demographic data of all surveyed primary care physicians and practices.

**Table 1:** Physicians characteristics (n = 715*)

		**n**	**%**
Age	<35	4	1
	35-49	276	39
	50-64	384	54
	>64	49	7
Gender	Male	455	64
	Female	252	35
Working time per week	up to 30	58	8
	31 to 40	105	15
	41 to 50	232	32
	51 to 60	220	31
	61 to 70	54	7
	71 to 99	36	5
Working experience in PC	<5	83	12
(years)	5 to 10	123	17
	11 to 20	240	34
	>20	251	35
Specialisation	GP/ Int.Med.	646	90
	Pediatrician	69	10

* n varies due to missing values.

**Table 2:** Practice characteristics (n = 715*)

		**n**	**%**
Number of physicians	1	373	52
	2	229	32
	3 to 5	95	13
	6 to 8	5	1
Number of non-physicians	0	21	3
	1 to 3	436	61
	4 to 6	196	27
	7 to 10	49	7
	>10	7	1
Location of practice	City	159	22
	Suburb	73	10
	Small town	272	38
	Rural area	193	27
Number of patients / week	up to 200	326	46
	201 to 400	314	44
	401 to 600	51	7
	601 to 900	5	1
Practice part of network	yes	125	18
	no	584	82

* n varies due to missing values.

**Figure 1:**
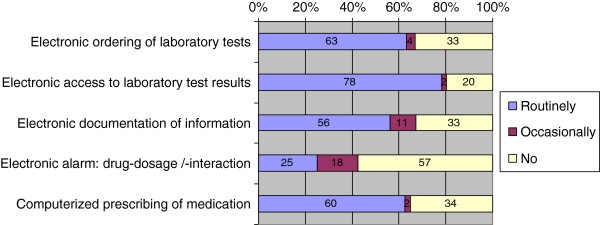
Use of certain electronic features and technologies by physicians.

As a result 497 (70%) primary care physicians in this survey use EMR. Routinely, 450 (63%) order laboratory tests electronically and 557 (78%) have electronic access to the laboratory results. Moreover 398 (56%) physicians routinely use electronic documentation of results and diseases, 233 (33%) do not at all. Electronic alarm functions for drug dosage or drug interaction were routinely used by 177 (25%) physicians, occasionally by 125 (18%) and not at all by 407 (57%). Furthermore 428 (60%) physicians were able to prescribe drugs with the use of a computer, but in contrast only 42 (6%) have the possibility to transfer prescriptions to a pharmacy electronically. Figure 1 shows the use of certain electronic features and technologies.

The currently used medical record system of surveyed practices enables 587 (82%) primary care physicians without major effort to generate a list of patients with a specified diagnosis, 320 (45%) can list their patients dependent on laboratory results. To extract a list of the current medication of patients is easy or somewhat difficult for 498 (70%) physicians, correlating to 481 (67%) who routinely provide a written list of medication to the patient. Additionally 431 (60%) of the physicians are able without major effort to generate a list of patients for whom tests or preventive care are due , but only 124 (17%) do routinely use a computerized recall system for patients with preventive or follow-up care needs (for example vaccinations or HbA1c testing in patients with diabetes). Similarly, only 85 (12%) physicians get computer-based reminders of guideline-based interventions or screening tests. Whereas 454 (64%) and 545 (76%) physicians do not use recall or reminders in patient care, respectively.

Only 10 (1%) physicians use email communication with patients for clinical or administrative purposes regularly, 424 (59%) never; even though 319 (45%) physicians estimate, that 1-9% of direct patient visitations of the last week could have been handled by phone or email contact with the patient. Out of the sample 51 (7%) physicians claim to get extra payment for non-personal interaction with patients, whereas 584 (82%) declare to get no additional fee.

When comparing gender and age groups in this survey, some differences could be seen. Male physicians were significantly more likely to use HIT (80%) compared to female physicians (64%). Furthermore, younger physicians (up to the age of 49 years) were significantly more likely to use electronic features like access to laboratory results or electronic documentation as well as regular email with patients.

**Figure 2:**
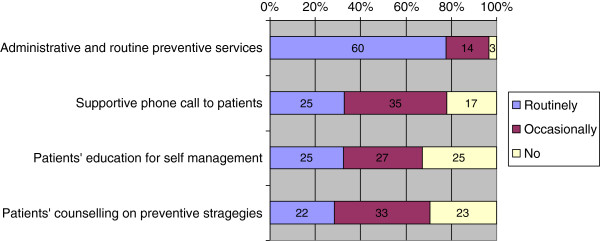
Task of nonphysician practice staff.

Altogether 516 (72%) primary care physicians declare that within their practice teams non-physician practice staff takes responsibility in patient care, 427 (60%) physicians claim to get support by non-physician practice staff in administrative services as ordering tests, writing prescriptions for long-term medication or in delivering routine preventive services. Furthermore 179 (25%) physicians report that their non-physician practice staff routinely calls patients to check on medications, symptoms and to help coordinate care in-between visits, 179 (25%) and 156 (22%) physicians, respectively, are supported by non-physician practice staff in patient education on self-management or counseling on prevention. Figure 2 gives an overview of tasks non-physician practice staff carries out in patient care. Additionally 112 (16%) physicians declare to get extra payment for specialization of non-physician practice staff, 554 (78%) claim they get no extra payment.

Regarding gender and age of primary care physicians, there were no significant differences in delegation of tasks to non-physician practice staff.

## Discussion

### Technical support of primary care physicians

Our results show that the majority of primary care physicians work with EMR in daily routine. Electronic features are mostly used to provide facilitation of administrative and routine work tasks such as electronic ordering and access to laboratory tests or electronic documentation of clinical information. Adequate HIT has the potential to improve practice management and quality of care [13,14]. Studies show advantages of paperless medical records [15], but also negative aspects of EMR as structural changes of the physician-patient-encounter for example by the position of the computer as well as reduced eye-to-eye contact and an increased duration of the visit are known [16,17].

The potential benefit of using electronic alarm functions for drug dosage or drug interaction is not fully utilized by physicians in this survey. There is evidence that computerized advice on drug dosage can improve the prescribing mode of medication. Often physicians complain about unnecessary or insufficient electronic alerts, this may be a reason for the low utilization of these functions. More research on design and context as well as how the advice should be delivered is needed to meet physicians’ needs [18,19].

While nearly two thirds of all surveyed primary care physicians are able to prescribe drugs computerized, only a minority is able to transfer prescriptions to a pharmacy electronically. In other countries, electronic prescribing, including electronic transfer of the prescription from the practice to a pharmacy, is enhanced through incentives by politics [20]. There is evidence that electronic prescribing of drugs can increase effective delivering of health care and patient safety by reducing medical errors in medication and can safe costs [21-23]. Studies show barriers towards electronic prescribing including interruption in work processes and security concerns. Therefore, practice and pharmacy transformation and redesign of work processes are required [24,25].

Only few physicians do routinely recall patients for preventive or follow-up care, although anticipatory and pro-active concepts in health care are essential [10-12]. Analogously, only a minority of physicians use computer-based reminders of guideline-based intervention or screening tests in daily routine. There is evidence that EMR reminders have an effect on provider behaviour regarding guideline or process adherence [26] and can improve medical outcome parameters [27]. Studies show that physicians appreciate electronic reminders for specific tasks if these alerts are accurate and do not disturb workflow [28]. In this analysis, a possible cause for the low utilization of recall or reminder in patient care may be a different understanding of these terms by physicians. In Germany, disease management programs for chronic diseases as diabetes or asthma are nationwide implemented since 2003. Recall of patients, for example for routine consultations or due examinations represents a core content of these programs. As the majority of physicians are nowadays enrolled in these programs, a higher rate of recalls should be assumed in reality for German primary care. Nevertheless, to guarantee adaption to users’ needs and to improve pro-active health care concepts, further research on current use, improvement and implementation of recall and reminder systems in primary health care in Germany is needed.

Yet another way to improve pro-active health care would be to support self-management of chronically ill patients by improving the utilization of ehealth, for example telemedical devices. In Germany, so far ehealth-systems are not routinely implemented nationwide. Studies exist, which show that patients in Germany do not know well about telemedical devices, but the majority approves the idea to use them in case of illness. Elderly approve less of ehealth, especially fearing the loss of personal contact with their physician [29]. How these issues have to be met, should be the content of further research.

Electronic communication via email with patients for clinical or administrative purposes is barely used by all surveyed physicians, even though they claim potential for this additional communication technology. Studies in other countries show that physicians and patients are willing to use this communication form for discussing different topics such as mostly non-acute medical symptoms, information about test results or administrative issues [30]. Physicians and patients claim advantages like saving time; concerns are expressed regarding security and confidentiality of emails. Some surveyed physicians declared they got extra financial compensation for non-personal communication via email or telephone between doctors and patients. Indeed, patients with a private health insurance can be billed for counseling by phone. Even in some cases of patients with a statutory health insurance, which cover 90% of German population, physicians can receive financial compensation for non-personal communication. But in general, no specific reimbursement or incentive for email communication between physicians and patients in Germany exists. Non-personal communication between non-physician practice staff and patients can not be billed at all. By introducing adequate financial compensation, the use of new communication technologies in patient care could be facilitated.

The significantly enhanced use of HIT and email with patients by younger and male primary care physicians could be explained by more common use of information technologies in younger generations and a greater affinity of men to use technical functions. It can be expected, that future generations of physicians as well as patients will have an increased demand for HIT and non-personal communication forms.

### Support of primary care physicians by practice staff

Over two thirds of primary care physicians get support in their daily work by sharing responsibility in patient care with non-physician practice staff. Whereas non-physician practice staff supports in administrative work and the delivery of routine preventive services, a more active role of non-physician practice staff in Germany is not common. Within other countries like Sweden or the United Kingdom, non-physician clinicians take responsibilities in patient care more often [5]. There is evidence that appropriately trained non-physician practice staff can ensure high quality in patient care and patient education [31-34]. In Germany, most primary care practices employ doctors’ assistants; only 6% of all practices employ a nurse [35], who are trained differently. Doctors’ assistants in Germany require additional education for taking more responsibility in patient care especially in aspects of medical care. An implementation of such education programmes has now been started [36,37]. Furthermore, the legal framework in Germany now allows physicians to delegate medical tasks like home visits, certain diagnostic examinations or special consultation hours for chronically ill patients to qualified non-physician practice staff. However, there is still a controversial discussion between stakeholders, politics and physicians in Germany about suitable tasks to delegate to non-physician practice staff [38]. It is important to consider the physicians’ perspective for future developments.

### Strengths

A strength of the study is the representative, randomly chosen sample of primary care physicians of all federal states of Germany. Therefore, the results can be interpreted as representative for Germany. The response rate of 49% was above average compared to the other surveyed countries and meets international standards in primary care [39,40]. No sociodemographic differences were identified between participants and non-responders.

### Limitations

In general, a questionnaire may be understood differently by surveyed persons. The computerized generation of certain lists of patient’ information was categorized into ‘easy’, ‘somewhat difficult’ and ‘difficult’ by the survey. Inter-personal rating differences should be considered when interpreting the results. Some questions could have been understood differently by the physicians. For example, the question “Do you use any of the following technologies in your practice: Electronic prescribing of medication?” does not explicitly elucidate whether a common way of computerized prescribing is used (computer-based generation of the prescription form, electronic fill-in by the physician, printing, validation by manual signature of the physician) or a fully electronic way of prescribing with electronic signature of the prescription and electronic transfer to the pharmacy which is not routinely used in Germany so far.

## Conclusion

German primary care physicians already get support in daily working routine by HIT and non-physician practice staff. However, the potential is not yet fully used. To increase the adoption of technical features like electronic alarm functions for medication or electronic prescribing, these should be technically improved and more adapted to physicians’ needs. Also, recall and reminder systems could be further developed to enable a more pro-active health care planning. New ways of communication via email could represent new forms of non-personal encounters of physicians and patients. Non-physician practice staff could play a more active role in health care. Patient education or counseling as well as routine telephone calls or home visits could be possible new working fields for doctors’ assistants in Germany. Therefore, adequate qualification of non-physician practice staff is mandatory and needs further development. Reimbursement should not only be linked to doctors’, but also to non-physician practice staff services. Primary care physicians should be more supported in daily routine to improve job satisfaction and to counteract the shortage of primary care physicians in the long-term.

## Competing interests

The authors declare that they have no competing interests.

## Authors’ contribution

AM, SJ and JSz conducted the German part of the CWF-survey in 2009. EU analyzed with AM and DO the data of the survey and wrote this publication. All authors made contribution to the manuscript and approved the final publication.

## Pre-publication history

The pre-publication history for this paper can be accessed here:

http://www.biomedcentral.com/1472-6947/12/81/prepub
